# Rapid broad-spectrum activity of molecular iodine against multidrug-resistant bacterial and fungal pathogens without residual staining

**DOI:** 10.1128/aac.00764-26

**Published:** 2026-09-09

**Authors:** Nandhana Nair, Arushi Sangal, Samuel Kim, Ilker Bayer, Victor Nizet, Elisabet Bjånes

**Affiliations:** 1 School of Biological Sciences, University of California San Diego 8784, La Jolla, California, USA; 2 I2Pure Corporation, Hudson, Massachusetts, USA; 3 Division of Host-Microbe Systems and Therapeutics, Department of Pediatrics, University of California San Diego 8784, La Jolla, California, USA; 4 Skaggs School of Pharmacy and Pharmaceutical Sciences, University of California 15500, La Jolla, California, USA; University of Iowa, Iowa City, Iowa, USA

**Keywords:** molecular iodine, disinfectant, multidrug-resistant pathogens, alternatives to antibiotics, environmental decontamination

## Abstract

Povidone iodine (PVP-I) is a widely used topical antiseptic with broad antimicrobial activity, but its clinical utility can be limited by skin irritation, slow drying, and persistent staining of skin, textiles, and surfaces. Here, we evaluated reformulated molecular iodine (I_2_) products as potential non-staining alternatives with retained or enhanced antimicrobial efficacy. Three I_2_ formulations (soak, nasal antiseptic, and hand ointment) were tested against 17 clinically relevant bacterial and fungal pathogens, including multidrug-resistant organisms and emerging nosocomial pathogens. Antimicrobial performance was assessed by quantitative kill assays, textile staining studies, and persistence testing on dried surfaces. All three I_2_ formulations demonstrated broad-spectrum activity against all organisms tested, with rapid 3–4 log_10_ reduction in colony-forming units (CFUs) or >99.9% killing commonly achieved within 15 s; selected fungal species required longer contact times or higher concentrations for maximal activity. Potent activity was observed against major healthcare-associated bacterial pathogens, including *Staphylococcus aureus*, *Enterococcus faecalis*, *Klebsiella pneumoniae*, and *Pseudomonas aeruginosa*, as well as fungal pathogens including *Candida albicans* and *Candidozyma auris*. Compared with PVP-I, I_2_ exhibited minimal residual staining and was readily removed from porous hospital textiles after brief water rinsing. Surface persistence studies further demonstrated sustained antimicrobial activity after drying. These findings indicate that I_2_ retains the broad-spectrum efficacy of traditional iodophors while offering practical advantages in material compatibility and reduced residual staining. I_2_ formulations may, therefore, represent useful alternatives for topical antisepsis and environmental decontamination in high-throughput healthcare settings where both antimicrobial performance and staining-related costs are important considerations.

## INTRODUCTION

Iodine is a broad-spectrum antiseptic with limited intrinsic aqueous solubility that has long been used in clinical settings for skin preparation, wound care, and procedural disinfection (1). In current practice, iodine is most commonly delivered as a complex with polyvinylpyrrolidone (povidone-iodine [PVP-I]), typically formulated as a 10% solution or 10,000 parts per million (PPM) containing approximately 1% available free iodine (I_2_). The antimicrobial activity of PVP-I derives from the gradual release of free I_2_, which penetrates microorganisms and oxidizes proteins, nucleotides, and membrane lipids, resulting in rapid cell death (2). This multitarget mechanism of action supports activity against a broad range of pathogens, including multidrug-resistant organisms, and may reduce the likelihood of acquired resistance, although intrinsic tolerance has been reported in some *Burkholderia* strains (3, 4).

Despite its broad utility, PVP-I has practical limitations. Repeated topical use may cause skin irritation or contact dermatitis in some individuals, and its characteristic brown-gold coloration can result in persistent staining of skin, textiles, and environmental surfaces. In healthcare environments, staining of gowns, linens, towels, and other porous materials may contribute to replacement costs, laundering burdens, and material waste. Molecular iodine (I_2_) formulations have emerged as potential alternatives designed to deliver high concentrations of active I_2_ while minimizing evaporative loss and avoiding carrier (5). Prior studies suggest that direct I_2_ exposure can be well tolerated even after repeated use and was not associated with significant toxicity in human skin adipose tissue explant models (6–8). Because these formulations primarily contain the active I_2_ species rather than a heterogeneous iodophor complex, they may offer efficient antimicrobial activity with improved material compatibility.

**Fig 1:**
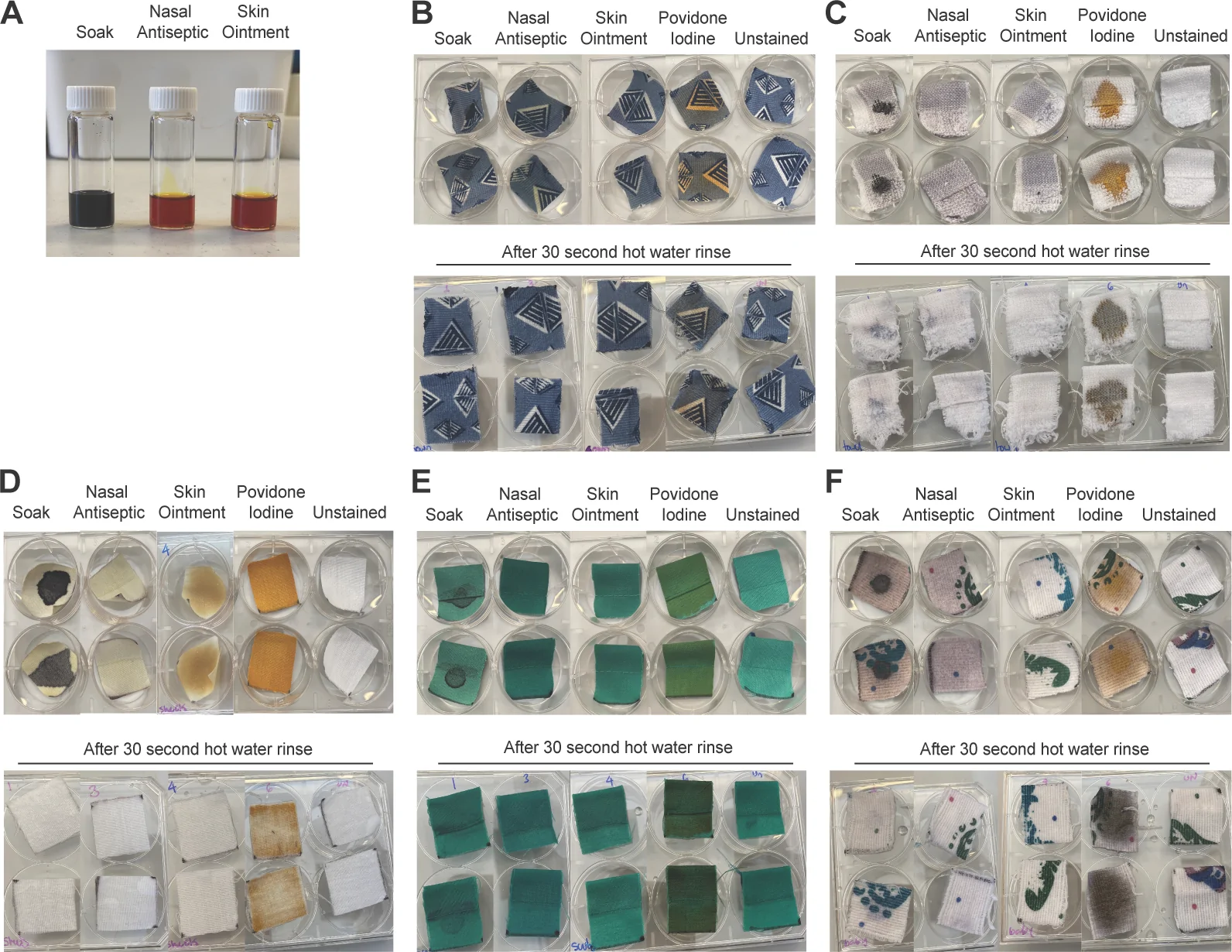
Molecular iodine does not stain hospital linens. (**A**) Molecular iodine products, soak, nasal antiseptic, and skin ointment. (**B through F**) Hospital linens were stained in duplicate with 100 μL of soak, nasal antiseptic, skin ointment, or povidone-iodine and allowed to dry for 4 days at room temperature. Linens were imaged, rinsed under hot running water for 30 s, and imaged again. (**B**) Gown, (**C**) towel, (**D**) sheets, (**E**) scrubs, and (**F**) baby linens. Representative of two independent experiments.

Here, we evaluated the antimicrobial, staining, and persistence properties of three I_2_ formulations: a soak, nasal antiseptic, and skin ointment (Fig. 1A). The activity was tested against clinically relevant bacterial and fungal pathogens, including healthcare-associated and multidrug-resistant organisms such as *Staphylococcus aureus*, *Enterococcus faecalis*, *Klebsiella pneumoniae*, *Pseudomonas aeruginosa*, and *Candida albicans* species*,* using quantitative microbicidal assays. We further assessed residual staining on hospital textiles and persistence of antimicrobial activity after drying on surfaces. All three I_2_ formulations demonstrated broad-spectrum activity against all organisms tested, with rapid killing frequently achieved within seconds of contact. In addition, the formulations were readily removed from hospital textiles after brief rinsing and retained antimicrobial activity after drying for up to 24 h. These findings support I_2_ as a potential practical alternative to PVP-I, for topical antisepsis and environmental decontamination, particularly in high-throughput healthcare settings where both antimicrobial performance and staining-related costs are important considerations.

## RESULTS

We first evaluated the staining properties of the I_2_ soak, nasal antiseptic, and skin ointment formulations on five different types of hospital linens, including a gown, towel, sheets, scrubs, and baby linens. Despite drying for 4 days, a 30-s rinse with hot water was sufficient for near-complete removal of all three I_2_ formulations (Fig. 1B through F). In contrast, PVP-I remained visibly retained on all five textiles after identical rinsing. A very slight residual stain from the soak formulation was observed on the towel, scrubs, and baby linens (Fig. 1C, E and F); however, this is expected to be removed with standard hospital laundering, which requires the use of water in excess of 71°C for at least 25 min along with the use of strong germicides (9). These results indicate that I_2_ lacks the persistent staining properties characteristic of PVP-I.

**Fig 2:**
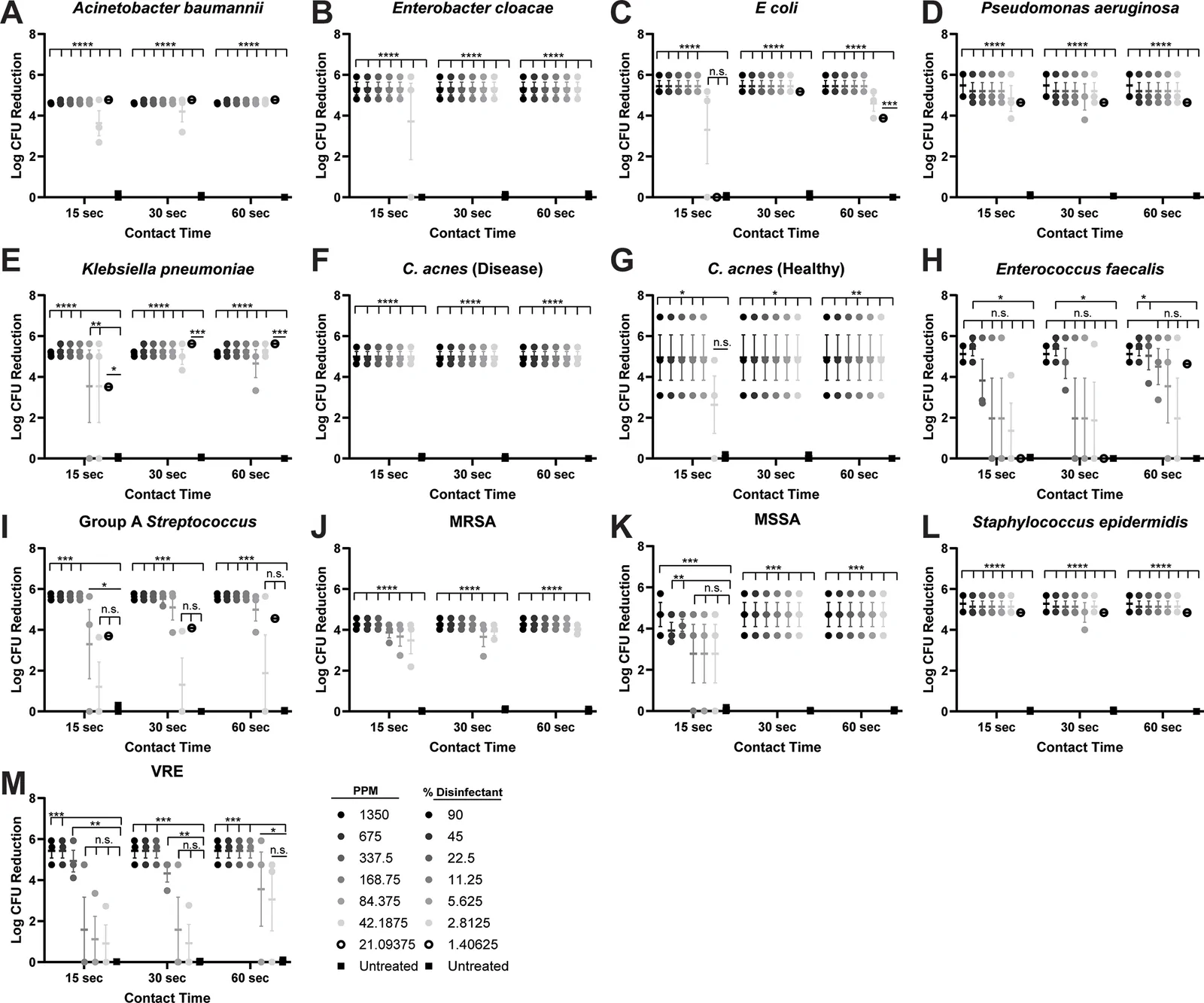
Iodine soak is highly effective at killing gram-negative and gram-positive pathogens. (**A through M**) Log CFU reduction after 15, 30, or 60 s contact time with iodine soak product. Iodine soak was serially diluted twofold in a 96-well plate, and ~ 10^5^ CFUs of washed bacterial suspension were added. After 15, 30, or 60 s, 50 mg/mL sodium thiosulfate was added to neutralize iodine. Wells were plated in triplicate for enumeration. All graphs are representative of technical replicates from three independent experiments ± SEM. Log CFU reduction for each group was compared to untreated by two-way analysis of variance (ANOVA). ****P < 0.0001, ***P < 0.001, **P < 0.01, and *P < 0.05; n.s. not significant. (**A through E**) Gram-negative bacterial pathogens (**A**) *Acinetobacter baumannii,* (**B**) *Enterobacter cloacae,* (**C**) *E. coli,* (**D**) *Klebsiella pneumoniae,* and (**E**) *Pseudomonas aeruginosa.* (**F through M**) Gram-positive pathogens **F**) *Cutibacterium acnes* (Disease), (**G**) *Cutibacterium acnes* (Healthy,) (**H**) *Enterococcus faecalis,* (**I**) Group A *Streptococcus,* (**J**) methicillin-resistant *Staphylococcus aureus,* (**K**) methicillin-sensitive *Staphylococcus aureus,* (**L**) *Staphylococcus epidermidis,* and (**M**) vancomycin-resistant *Enterococcus faecalis.*

**Fig 3:**
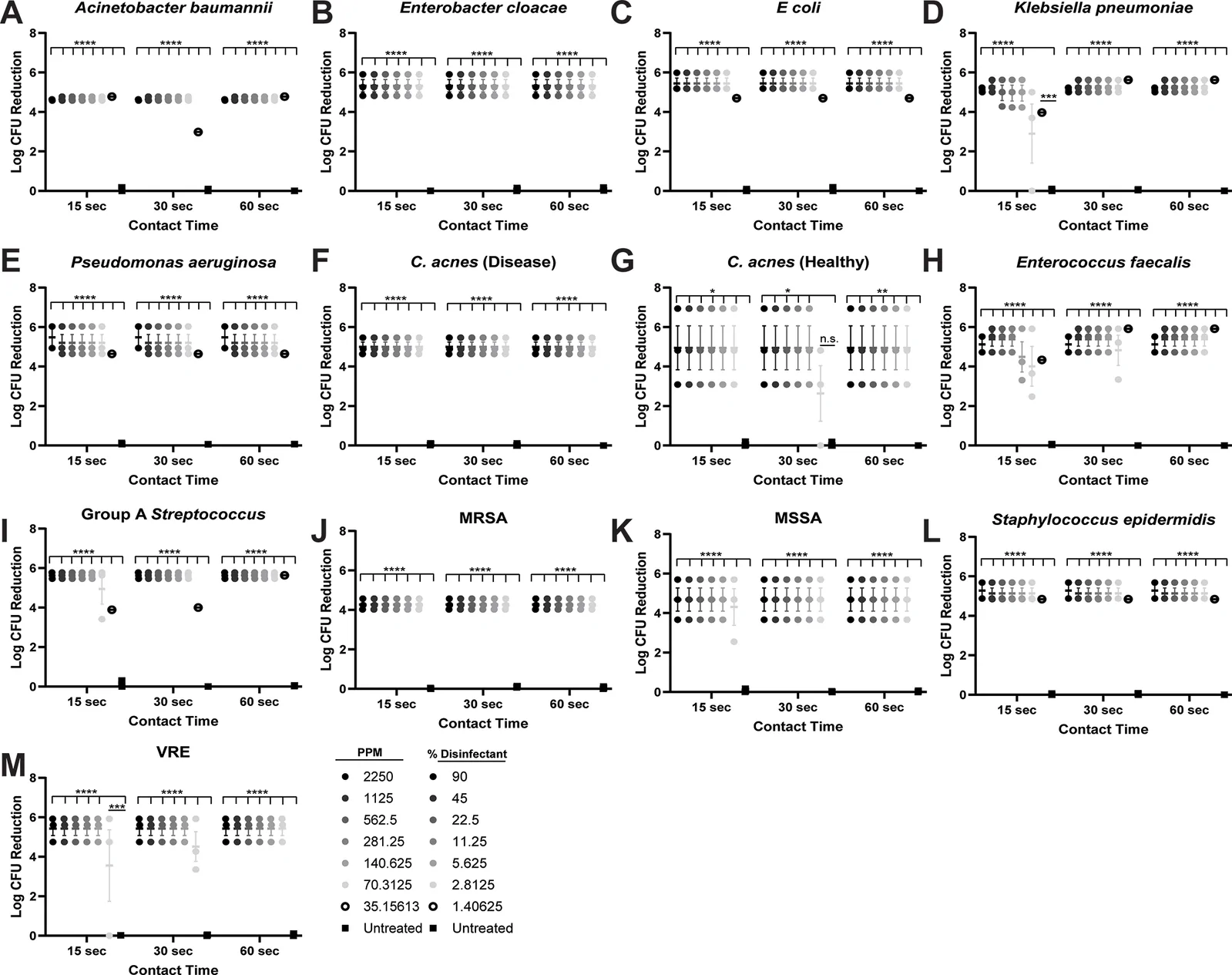
Iodine nasal antiseptic is highly effective at killing gram-negative and gram-positive pathogens. (**A through M**) Log CFU reduction after 15, 30, or 60 s contact time with iodine nasal antiseptic. Iodine nasal antiseptic was serially diluted twofold in a 96-well plate, and ~ 10^5^ CFUs of the washed bacterial suspension were added. After 15, 30, or 60 s, 50 mg/mL sodium thiosulfate was added to neutralize iodine. Wells were plated in triplicate for enumeration. All graphs are representative of technical replicates from three independent experiments ± SEM. Log CFU reduction for each group was compared to untreated by two-way ANOVA. ****P < 0.0001, ***P < 0.001, **P < 0.01, and *P < 0.05; n.s. not significant. (**A through E**) Gram-negative bacterial pathogens (**A**) *Acinetobacter baumannii,* (**B**) *Enterobacter cloacae,* (**C**) *E. coli,* (**D**) *Klebsiella pneumoniae,* and (**E**) *Pseudomonas aeruginosa.*(**F through M**) Gram-positive pathogens (**F**) *Cutibacterium acnes* (Disease), (**G**) *Cutibacterium acnes* (Healthy), (**H**) *Enterococcus faecalis,* (**I**) Group A *Streptococcus,* (**J**) methicillin-resistant *Staphylococcus aureus,* (**K**) methicillin-sensitive *Staphylococcus aureus,* (**L**) *Staphylococcus epidermidis,* and **M**) vancomycin-resistant *Enterococcus faecalis.*

We next evaluated the antimicrobial activity of the I_2_ soak formulation against 13 gram-negative and gram-positive bacterial species (Fig. 2; Fig. S1). The soak formulation demonstrated broad-spectrum activity, with 3–4 log_10_-fold reductions in CFUs observed in nearly all strains after 15 s of contact time at concentrations as low as 20–40 ppm (1.4%–2.8%). Group A *Streptococcus* (GAS), *Enterococcus faecalis*, and vancomycin-resistant *Enterococcus faecalis* (VRE) required longer contact times or higher concentrations for maximal killing (Fig. 2H, I and M). These findings are consistent with reported minimum bactericidal concentrations (MBCs) for 10% PVP-I (10–13). Consistent with these findings, the nasal antiseptic formulation demonstrated similar potent activity, achieving >4 log_10_-fold reduction in nearly all bacterial species within 15 s (Fig. 3; Fig. S2). The nasal antiseptic was modestly more effective than the iodine soak against *E. faecalis,* Group A *Streptococcus,* and VRE (Fig. 2H, I and M).

**Fig 4:**
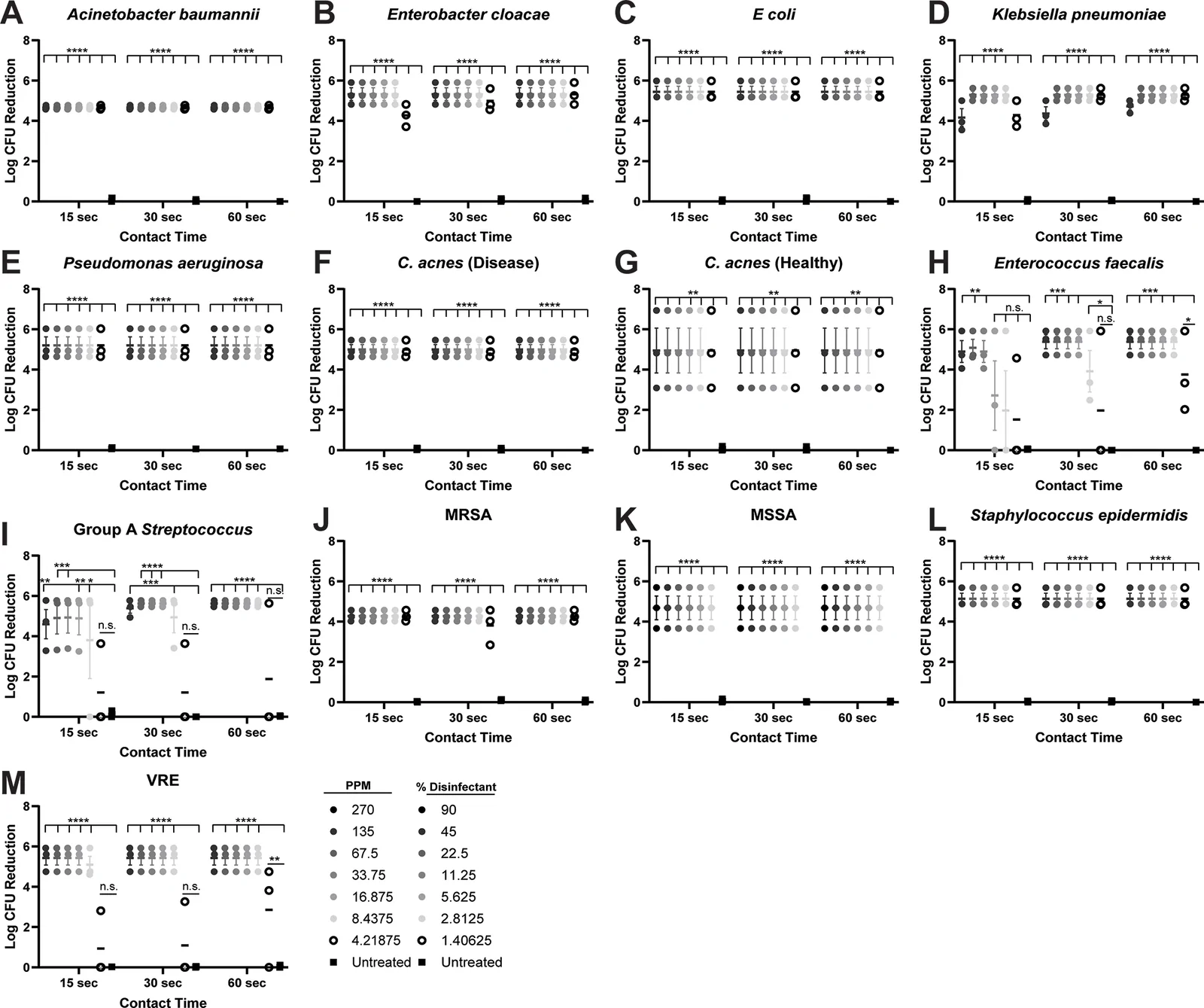
Iodine skin ointment is highly effective at killing gram-negative and gram-positive pathogens. (**A through M**) Log CFU reduction after 15, 30, or 60 s contact time with iodine skin ointment. Iodine skin ointment was serially diluted twofold in a 96-well plate, and ~ 10^5^ CFUs of the washed bacterial suspension were added. After 15, 30, or 60 s, 50 mg/mL sodium thiosulfate was added to neutralize iodine. Wells were plated in triplicate for enumeration. All graphs representative of technical replicates from three independent experiments ± SEM. Log CFU reduction for each group was compared to untreated by two-way ANOVA. ****P < 0.0001, ***P < 0.001, **P < 0.01, and *P < 0.05; n.s. not significant. (**A through E**) Gram-negative bacterial pathogens **A**) *Acinetobacter baumannii,* (**B**) *Enterobacter cloacae,* (**C**) *E. coli,* (**D**) *Klebsiella pneumoniae,* and (**E**) *Pseudomonas aeruginosa.* (**F through M**) Gram-positive pathogens (**F**) *Cutibacterium acnes* (Disease), (**G**) *Cutibacterium acnes* (Healthy), (**H**) *Enterococcus faecalis,* (**I**) Group A *Streptococcus,* (**J**) methicillin-resistant *Staphylococcus aureus,* (**K**) methicillin-sensitive *Staphylococcus aureus,* (**L**) *Staphylococcus epidermidis,* and (**M**) vancomycin-resistant *Enterococcus faecalis.*

The skin ointment formulation exhibited comparable antibacterial activity to both the soak and nasal antiseptic, with 3–4 log_10_-fold reductions observed after 15 s (Fig. 4; Fig. S3). GAS, *E. faecalis*, and VRE displayed time- and concentration-dependent killing, with maximal efficacy at 15 s and approximately 67.5 ppm. Notably, the skin ointment is formulated at a lower starting concentration of I_2_, yet it remains highly effective against multiple strains, including when diluted to <5 ppm. Greater variability in CFU reduction was observed at earlier time points across several strains.

**Fig 5:**
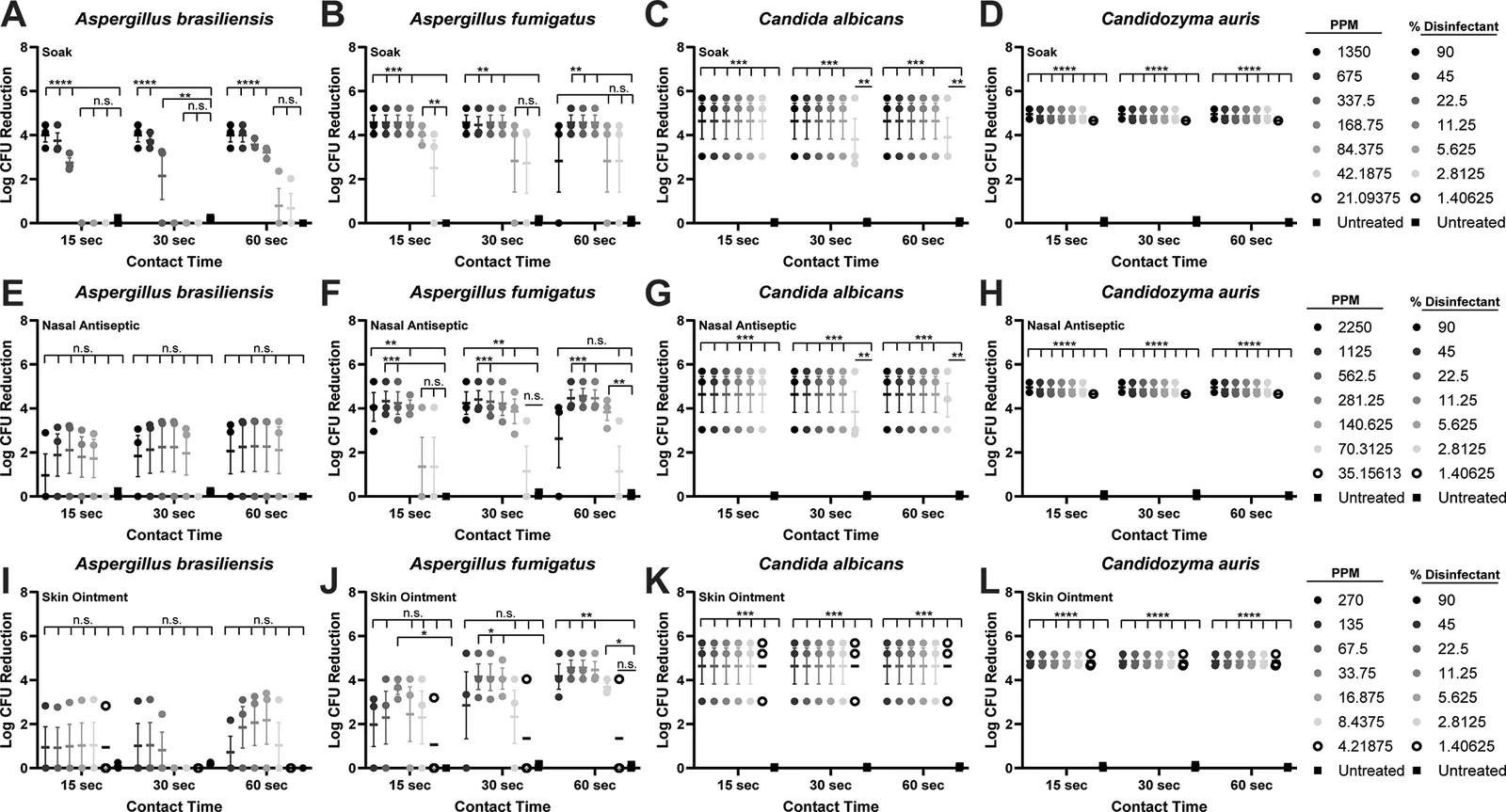
Iodine hand ointment is highly effective at killing fungal pathogens. (**A through L**) Log CFU reduction after 15, 30, or 60 s contact time with iodine soak, nasal antiseptic, and skin ointment. Iodine products were serially diluted twofold in a 96-well plat, and ~ 10^5^ CFUs of the washed fungal suspension were added. After 15, 30, or 60 s, 50 mg/mL sodium thiosulfate was added to neutralize iodine. Wells were plated in triplicate for enumeration. All graphs are representative of technical replicates from three independent experiments ± SEM. Log CFU reduction for each group was compared to untreated by two-way ANOVA. ****P < 0.0001, ***P < 0.001, **P < 0.01, and *P < 0.05; n.s. not significant. (**A through D**) Iodine soak. (**E through H**) Nasal antiseptic. (**I through L**) Skin ointment. (**A, E, and I**) *Aspergillus brasiliensis.* (**B, F, and J**) *Aspergillus fumigatus.* (**C, G, and K**) *Candida albicans.* **D, H, and L**) *Candidozyma auris.*

We next investigated antifungal activity against clinically relevant environmental fungal species. *Candida albicans* and *Candidozyma auris* (formerly known as *Candida auris*) are increasingly recognized nosocomial pathogens, particularly in immunocompromised patients (14–16). *Aspergillus fumigatus* and *Aspergillus brasiliensis* are ubiquitous environmental fungi capable of causing opportunistic infections in highly immunocompromised individuals (17) and are commonly used in industrial and regulatory settings as standard quality control microorganisms. The I_2_ soak formulation achieved rapid killing of both *Aspergillus* species at high concentrations (>300 ppm) within 15 s (Fig. 5A and B). In contrast, the nasal antiseptic and skin ointment formulations required higher concentrations and longer contact times, with significant CFU reductions observed against *A. fumigatus* at 60 s. Neither the nasal antiseptic nor skin ointment was effective against *A. brasiliensis* at the tested time points. Previous reports demonstrate that PVP-I requires a contact time of 5–10 min for fungal killing (18). With longer contact times, it is possible that the antiseptic and hand ointment would achieve efficacy against *A. brasiliensis*. All three I_2_ formulations demonstrated strong activity against *C. albicans* and *C. auris*, with rapid killing observed at low concentrations (Fig. 5).

**Fig 6:**
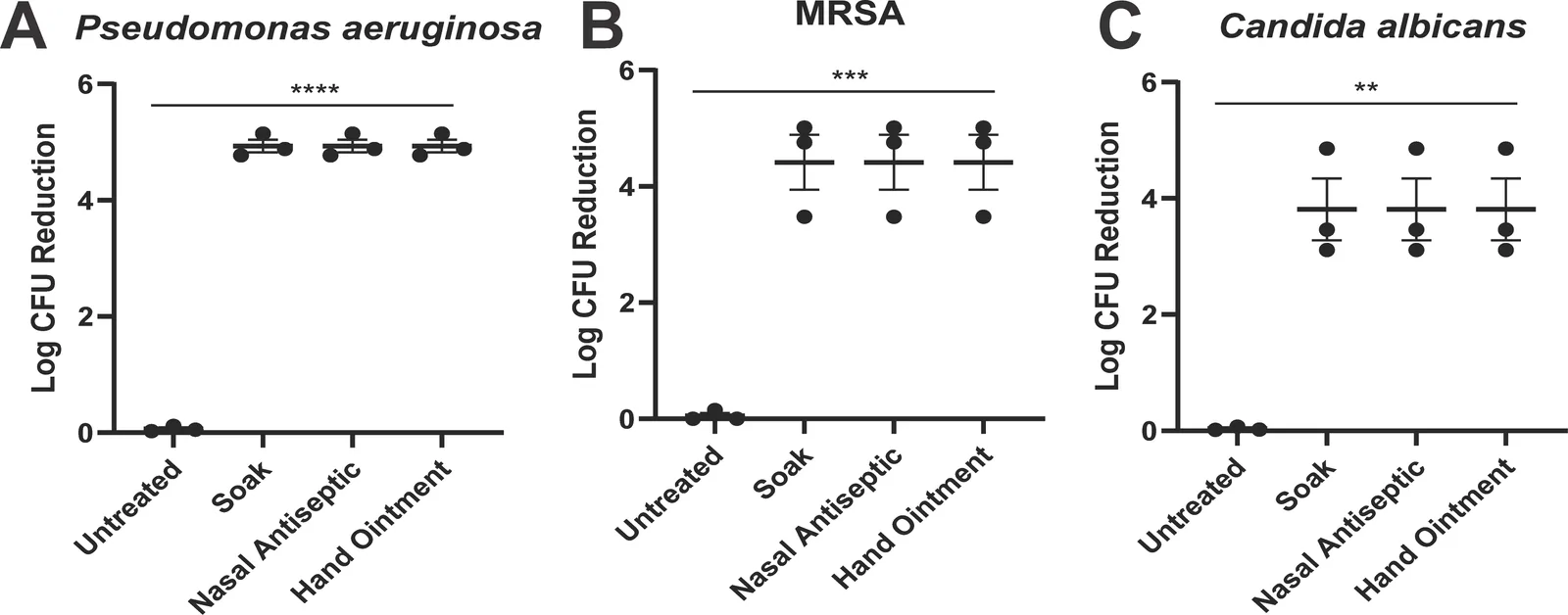
Iodine products have persistent antimicrobial activity. Fifty microliters of undiluted soak, nasal antiseptic, or hand ointment was added onto 1 cm^2^ filter paper coupons and allowed to dry for 24 h. ~10^5^ CFUs of (**A**) *Pseudomonas aeruginosa*, (**B**) MRSA, or (**C**) *Candida albicans* were added in triplicate in PBS and incubated with gentle agitation for 1 min. Conditions were immediately serially diluted and plated for enumeration. All graphs are representative of technical replicates from three independent experiments ± SEM. Log CFU reduction for each group was compared to untreated by one-way ANOVA. ****P < 0.0001, ***P < 0.001, **P < 0.01, and *P < 0.05; n.s. not significant.

**Fig 7:**
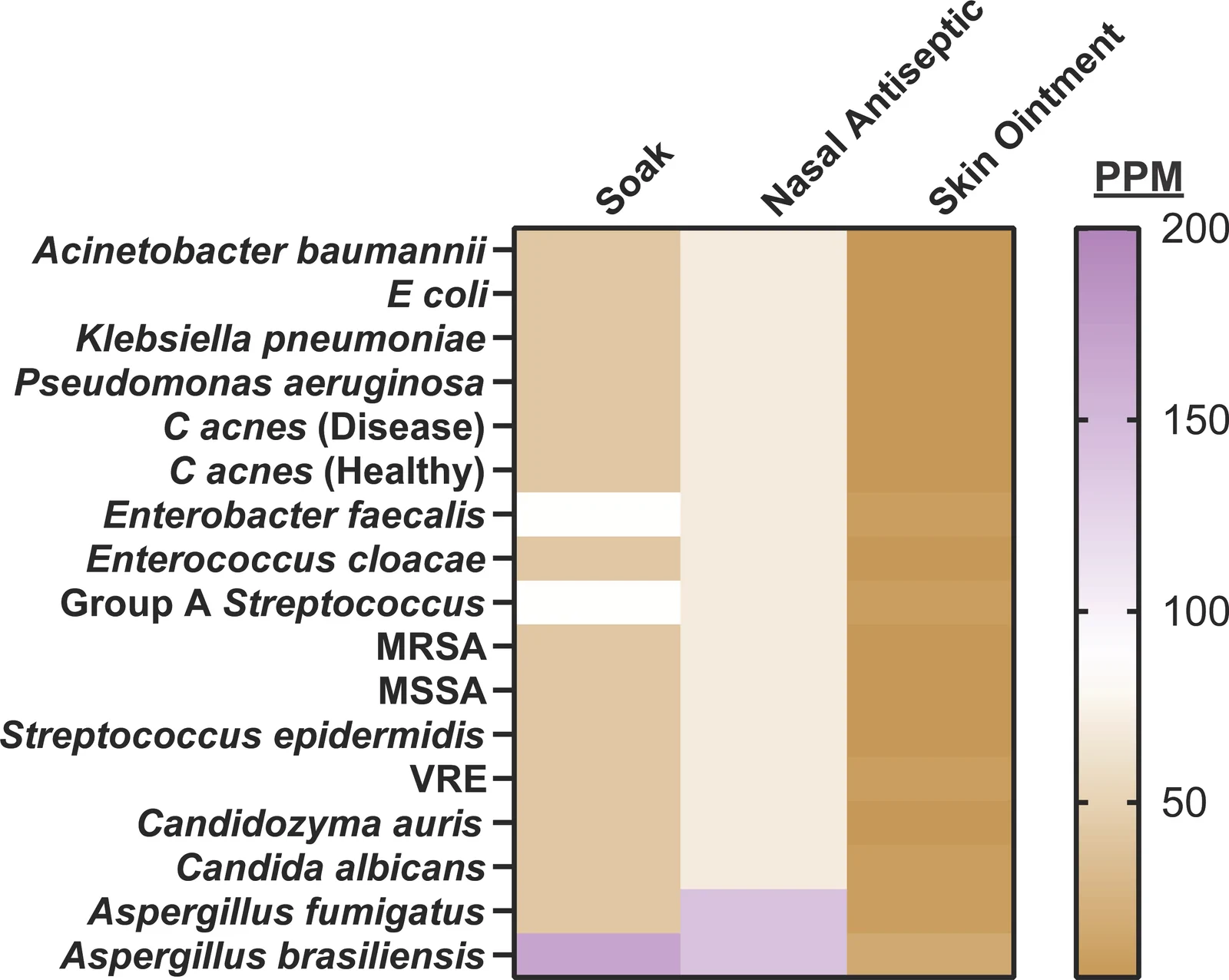
Iodine soak, nasal antiseptic, and skin ointment are highly effective antibacterial and antifungal agents. Heatmap summary of minimum bactericidal or fungicidal (MBC and MFC) concentration in parts per million (PPM) required to kill >99.9% of the starting inoculum for all strains tested. Exact values are provided in Table S1.

Finally, we evaluated the persistence of antimicrobial activity following drying on surfaces, an important property for infection control. I_2_ soak, nasal antiseptic, and skin ointment formulations were applied to filter paper and allowed to dry for 24 h prior to microbial challenge with *Pseudomonas aeruginosa*, MRSA, or *Candida albicans*. All three formulations retained substantial antimicrobial activity after drying, with near-complete CFU reduction observed across all tested organisms (Fig. 6). Collectively, these results demonstrate that I_2_ formulations exhibit rapid, broad-spectrum antimicrobial activity against bacterial and fungal pathogens, including multidrug-resistant strains, while also maintaining persistent activity after drying and minimizing staining of hospital textiles (Fig. 7).

## DISCUSSION

Due to the limited stability and solubility of free iodine, antiseptic formulations have historically relied on iodophores such as PVP, which act as reservoirs that gradually release biocidal I_2_. The antimicrobial activity of PVP-I depends on equilibrium-driven release of free iodine, with maximal available I_2_ achieved at intermediate dilutions (10, 19). This counterintuitive release is known as the dilution paradox where free iodine reaches maximal levels at ~20 ppm as PVP-I is diluted to ~0.1% with a dynamic equilibrium between iodine species (19, 20). Since free iodine is highly unstable, complexing with PVP is typically used to improve stability. While dilution can increase tolerability, it may also constrain the delivery of active iodine at higher concentrations. In this study, the evaluated formulations were designed to provide a stable and consistent source of I_2_ without reliance on a carrier reservoir, thereby enabling direct delivery of active iodine. This approach may permit higher effective concentrations of I_2_ at the site of application and contribute to rapid antimicrobial activity observed across all three formulations.

PVP-I and chlorhexidine remain among the most widely used antiseptics in clinical practice (21). However, both agents have practical limitations. PVP-I is associated with persistent dark golden staining of skin, teeth, textiles, and surfaces, and chlorhexidine can also leave visible residue and has been associated with rare but significant adverse reactions. In healthcare environments, staining of linens and materials contributes to increased laundering burden, material turnover, higher operating costs, and reduced sustainability measures (22). Consistent with prior reports that I_2_ does not stain human skin or tissue explants (6), we observed that all three I_2_ formulations were readily removed from hospital textiles with brief water rinsing. This lies in stark contrast to persistent staining by PVP-I that requires specialized detergents and chemicals, which are often only partially effective. A limitation of these findings is that only fabrics made of cotton and polyester were tested. Nevertheless, our findings suggest that I_2_ formulations may offer practical advantages in settings where material compatibility and turnover are important considerations. Future studies should evaluate staining and compatibility across a broader range of clinical surfaces and instruments, as well as potential applications in oral and dental settings where PVP-I-associated staining remains a limitation (23, 24).

Across all three formulations, I_2_ demonstrated rapid, broad-spectrum antimicrobial activity against bacterial and fungal pathogens, including multidrug-resistant organisms. Greater than 99.9% killing was frequently achieved within 15 s for most bacterial species including major healthcare-associated pathogens such as MRSA, VRE, *K. pneumoniae*, and *P. aeruginosa*. Importantly, the three I_2_ formulations were also highly effective against emerging fungal pathogens such as *Candida albicans* and *Candidozyma auris*, with >99.9% killing observed in 15 s. While higher concentrations and longer contact times were required for maximal killing of *Aspergillus* spp., this is consistent with previously reported kinetics for PVP-I and other antiseptics against these fungi (10–13, 25, 26). One limitation of this study is that only 17 microorganisms were tested. Future studies will examine additional pathogens along with clinical isolates.

Persistence of antimicrobial activity is an important feature for infection control. We observed that I_2_ formulations retained substantial activity after drying, with near-complete killing of representative bacterial and fungal pathogens following 24 h of surface exposure. This contrasts with concerns regarding reduced efficacy or emerging tolerance associated with repeated exposure to certain antiseptics. For example, a recent study swabbed over 200 high-touch surfaces in an ICU and identified chlorhexidine tolerance among recovered bacteria, including both recognized nosocomial pathogens and environmental contaminants (27). The authors linked this tolerance to residual chlorhexidine found on common hospital surfaces more than 24 h after application, even after routine cleaning. In contrast, repeated exposure to iodine has not been associated with the development of clinically significant resistance, likely reflecting its multitarget oxidative mechanism. However, contamination of non-sterile iodine preparations with intrinsically tolerant organisms such as *Burkholderia cepacia* has been reported and can lead to infection following clinical use (3, 4, 28). These observations highlight the importance of formulation integrity and appropriate handling rather than true antimicrobial resistance. While the need for effective decontamination of clinical environments, wounds, and surgical sites remains paramount, these findings underscore the importance of proper product formulation and use to minimize unintended microbial exposure.

Collectively, our findings demonstrate that I₂ formulations achieve rapid microbial reduction across multiple bacterial and fungal pathogens, including the leading clinically relevant organisms, with substantial killing observed within short contact times. In addition to antimicrobial activity, I_2_ formulations were readily removed from hospital textiles with minimal residual staining and retained activity following drying on surfaces for up to 24 h. These combined properties distinguish I₂ from traditional iodophores and support its potential utility as an alternative for topical antisepsis and environmental decontamination, particularly in healthcare settings where rapid action, material compatibility, and operational efficiency are critical.

## MATERIALS AND METHODS

### Bacterial and fungal methods

All strains utilized are listed in Table 1.

**TABLE 1:** Bacterial and fungal strains

Gram-negative species	Strain ID	Source
*Acinetobacter baumannii*	LAC-4	(29)
*Escherichia coli*	CFT073	(30)
*Klebsiella pneumoniae*	NTUH K2044	(30, 31)
*Pseudomonas aeruginosa*	PA14	(30–32)

Gram-positive species	Strain ID	Source
*Cutibacterium acnes* (associated with healthy skin)	HL110PA3	(33)
*Cutibacterium acnes* (associated with acne)	HL043PA1	(33)
*Enterobacter cloacae*	ATCC 35030	ATCC
*Enterococcus faecalis*	ATCC 29212	ATCC
Group A *Streptococcus*	M1 5448	(34)
Methicillin-resistant *Staphylococcus aureus* (MRSA)	TCH1516	(34, 35)
Methicillin-sensitive *Staphylococcus aureus* (MSSA)	ATCC 29213	ATCC
*Staphylococcus epidermidis*	ATCC 1457	ATCC
Vancomycin-resistant *Enterococcus faecalis*	ATCC 51299	ATCC

Fungal species	Strain ID	Source
*Aspergillus brasiliensis*	ATCC 16404	ATCC
*Aspergillus fumigatus*	9.25	Clinical isolate provided by Stephanie Fraley. (36)
*Candida albicans*	SC5314 (ATCC MYA-2876)	ATCC
*Candidozyma auris*	381	CDC AR Isolate Bank

### Bacterial cultures

*Acinetobacter baumannii*, *Escherichia coli, Klebsiella pneumoniae,* and *Pseudomonas aeruginosa* were grown to the stationary phase in Luria Broth at 37°C with aeration. *Cutibacterium acnes* was grown on tryptic soy agar with 5% sheep’s blood (ThermoFisher) or in brain heart infusion (BHI) in a sealed plastic box at 37°C using a GasPak anaerobic system. Cultures were grown to the stationary phase for 4–5 days. *Enterobacter cloacae, Enterococcus faecalis,* methicillin-resistant and methicillin-sensitive *Staphylococcus aureus* (MRSA and MSSA), *Staphylococcus epidermidis*, and vancomycin-resistant *Enterococcus faecalis* (VRE) were grown to the stationary phase in Todd-Hewitt broth (THB) at 37°C with aeration. Group A *Streptococcus* was grown to the stationary phase in THB at 37°C without aeration. Stationary-phase cultures were washed twice with 1× PBS, resuspended to OD_600_ ~0.4, and diluted 1:10 or 1:100 in sterile PBS to obtain ~1 × 10^7^ CFUs/mL. Ten microliters of the bacterial suspension was added to triplicate wells for microbicidal assays (~1 × 10^5^ CFUs/well).

### Fungal cultures

*Candida albicans* and *Candidozyma auris* were grown in yeast extract peptone dextrose (YPD) broth to the stationary phase at 30°C with aeration. Plates were incubated at 30°C for 24–36 h. Cultures were washed twice with 1× PBS, resuspended to OD_600_ ~0.4, and diluted 1:10 in sterile PBS to obtain ~1 × 10^7^ CFUs/mL. *Aspergillus fumigatus* and *Aspergillus brasiliensis* were grown in YPD or malt extract agar at 37°C without aeration. Spores were collected using a sterile loop premoistened in 0.2% Triton to disperse spores. Spore suspensions were normalized to OD_600_ ~0.4 (~1 × 10^7^ CFUs/mL). *A. brasiliensis* solutions were diluted 1:10 in sterile PBS as needed. Ten microliters of fungal suspensions (~1 × 10^5^ CFUs/well) was added to triplicate wells.

### Molecular iodine (I₂) formulations

Proprietary solutions of one or more of the following components were used as a rheology modifier: elemental iodine, starch, sodium iodide, propylene glycol, isopropyl alcohol, vegetable glycerin, and hydroxypropyl methylcellulose (HPMC). Each component was purchased from Merck (USA). High-purity deionized (DI) water was utilized as the aqueous medium for all preparation and dilution steps.

### Preparation of iodine-based formulations

Three distinct iodine-based antimicrobial solutions were prepared using varied delivery vehicles and stabilization methods, with iodine concentrations verified to ensure precise parts-per-million (ppm) quantities.

*Modified starch-iodine suspension (iodine soak*). An aqueous starch suspension in DI water was prepared to facilitate optimal iodine complexation. The resulting suspension was iodinated with iodine topical solution (a combination of iodine and sodium iodide) to a titratable iodine concentration of 1,500 ppm. Sodium iodide (NaI) and glycerin were incorporated as inactive stabilizers to prevent iodine hydrolysis and maintain long-term stability.

*Propylene glycol-based nasal antiseptic*. A non-aqueous nasal antiseptic was formulated using propylene glycol as the primary carrier. Iodine topical solution (a combination of iodine and sodium iodide) was dispersed into the propylene glycol to a titratable iodine concentration of 2,500 ppm. Sodium iodide (NaI) was added to the formulation as a chemical stabilizer.

*Iodinated isopropyl alcohol hand sanitizer* (skin ointment). An alcohol-based hand sanitizer was synthesized by combining 70% (vol/vol) isopropyl alcohol and DI water. The solution was iodinated to a titratable iodine concentration of 300 ppm. To improve application characteristics and increase contact time, a desired viscosity was achieved via incorporation of HPMC.

### Killing assays

To assess microbicidal activity, I_2_ formulations (soak, nasal antiseptic, and skin ointment) were serially diluted two-fold in PBS in 96-well round-bottom plates. Ten microliters of bacterial or fungal suspension (~1×10^5^ CFUs/well) was added to each well. After 15, 30, or 60 s, 10 µL of a 10× stock of 50 mg/mL sodium thiosulfate (Sigma) was added to neutralize active I_2_ (37 and mixed until the solution became colorless. Samples were spot-plated in triplicate onto appropriate agar plates and incubated at 30°C or 37°C. Plates were imaged and colonies enumerated. Untreated controls were plated with and without sodium thiosulfate to confirm input CFUs and validate neutralization without growth inhibition. The limit of detection was 10 CFU (1 CFU per plate × plating factor 10). Log CFU reduction was calculated relative to input CFUs. Percent CFU reduction was calculated as (plated CFU/input CFU) × 100. Minimum bactericidal or fungicidal concentration (MBC and MFC) was defined as the lowest concentration achieving >99.9% killing according to Clinical Laboratory Standards Institute (CLSI) guidelines.

### Persistence assays

Fifty microliters of the I_2_ formulations (soak, nasal antiseptic, or skin ointment) was applied in triplicate to 1-cm^2^ filter paper coupons and allowed to dry at room temperature overnight. The paper coupons were not protected from light to mimic real-world application. Forty microliters of *P. aeruginosa*, MRSA, or *Candida albicans* (~10^5^ CFU) was added with 360 µL of sterile PBS and incubated with gentle agitation for 1 min. Samples were serially diluted in PBS, plated on appropriate agar plates, and incubated at 30°C or 37°C. Untreated paper coupons were used to determine input CFUs. The limit of detection was 40 CFU (1 CFU per plate × plating factor 40). Log and percent CFU reduction were calculated as described above.

### Staining assays

Clean hospital linens were obtained from Emerald Textiles (UC San Diego healthcare system supplier). One hundred microliters of I2 formulations (soak, nasal antiseptic, and skin ointment) or povidone-iodine (CVS, 10% solution, 10,000 ppm) was applied to 1-inch^2^ swatches cut from baby linens (100% cotton), scrubs (55% cotton, 45% polyester), a towel (74% cotton, 26% polyester), sheets (55% cotton, 45% polyester), and a hospital gown (55% cotton, 45% polyester) and allowed to dry for 4 days at room temperature. Stained linen swatches were rinsed under hot running tap water (~37°C) for 30 s with gentle abrasion. Unstained swatches served as controls.

### Statistical analysis

Statistical analysis was performed using GraphPad Prism v11. Comparisons to untreated controls were performed using two-way analysis of variance (ANOVA) with Dunnett’s multiple comparison post-test. Persistence assays were analyzed by one-way ANOVA. Unless otherwise indicated in figure legends, data represent the means ± standard error of the mean (SEM) from three or more independent biological replicates. *P* < 0.05 was considered significant.

## Data Availability

All data are provided in Data S1.

## SUPPLEMENTAL MATERIAL

Data S1 aac.00764-26-s0001.xlsx Contains data included in manuscript.

Supplemental material aac.00764-26-s0002.docx Fig. S1 to S4; Table S1.

ASM does not own the copyrights to Supplemental Material that may be linked to, or accessed through, an article. The authors have granted ASM a non-exclusive, world-wide license to publish the Supplemental Material files. Please contact the corresponding author directly for reuse.
