# Supplementary material for: Rapid broad-spectrum activity of molecular iodine against multidrug-resistant bacterial and fungal pathogens without residual staining
Source: Antimicrob Agents Chemother. 2026 Sep 9;70(10):e00764-26. doi: 10.1128/aac.00764-26 (PMC13689429; doi:10.1128/aac.00764-26)
Supplement: Supplementary file 1 [file aac.00764-26-s0002.docx]

**Supplemental Data File**

**Killing without a trace: Molecular iodine efficacy against clinically relevant bacterial and fungal pathogens**

Arushi Sangal^1☨^, Nandhana Nair^1☨^, Samuel Kim^1^, Ilker Bayer^2^, Victor Nizet^3,4^*, Elisabet Bjånes^3^*

^1^School of Biological Sciences, University of California San Diego, La Jolla, CA, USA

^2^I_2_Pure Corporation, Hudson, MA, USA

^3^Division of Host-Microbe Systems and Therapeutics, Department of Pediatrics, University of California San Diego, La Jolla, CA, USA

^4^Skaggs School of Pharmacy and Pharmaceutical Sciences, University of California, La Jolla, California, USA

***Correspondence:**

Corresponding Author

[vnizet@ucsd.edu](mailto:vnizet@ucsd.edu), [ebjanes@ucsd.edu](mailto:ebjanes@ucsd.edu)

**Contents:**

Figure 1: Iodine soak is highly effective at killing Gram-negative and Gram-positive pathogens.

Figure 2: Iodine nasal antiseptic is highly effective at killing Gram-negative and Gram-positive pathogens.

Figure 3: Iodine skin ointment is highly effective at killing Gram-negative and Gram-positive pathogens.

Figure 4: Iodine disinfectants are highly effective at killing fungal pathogens.

Table 1: Minimum bactericidal concentrations for killing bacterial and fungal pathogens.

**Supplemental Figure 1: Iodine soak is highly effective at killing Gram-negative and Gram-positive pathogens.** A-M) Percent CFU reduction after 15, 30, or 60 seconds contact time with iodine soak product. Iodine soak was serially diluted two fold in a 96 well plate and ~10^5^ CFUs of washed bacterial suspension were added. After 15, 30, or 60 seconds, 50 mg/mL sodium thiosulfate was added to neutralize iodine. Wells were plated in triplicate for enumeration. Percent CFU calculated relative to untreated conditions. All graphs representative of technical replicates from three independent experiments ± SEM. A-E) Gram-negative bacterial pathogens A) *Acinetobacter baumannii* B) *Enterobacter cloacae* C) *E. coli* D) *Klebsiella pneumoniae* E) *Pseudomonas aeruginosa* F-M) Gram-positive pathogens F) *Cutibacterium acnes* (Disease) G) *Cutibacterium acnes* (Healthy) H) *Enterococcus faecalis* I) Group A *Streptococcus* J) Methicillin resistant *Staphylococcus aureus* K) Methicillin sensitive *Staphylococcus aureus* L) *Staphylococcus epidermidis* M) vancomycin resistant *Enterococcus faecalis.*

**Supplemental Figure 2: Iodine nasal antiseptic is highly effective at killing Gram-negative and Gram-positive pathogens.** A-M) Percent CFU reduction after 15, 30, or 60 seconds contact time with iodine nasal antiseptic. Iodine nasal antiseptic was serially diluted two fold in a 96 well plate and ~10^5^ CFUs of washed bacterial suspension were added. After 15, 30, or 60 seconds, 50 mg/mL sodium thiosulfate was added to neutralize iodine. Wells were plated in triplicate for enumeration. Percent CFU calculated relative to untreated conditions. All graphs representative of technical replicates from three independent experiments ± SEM. A-E) Gram-negative bacterial pathogens A) *Acinetobacter baumannii* B) *Enterobacter cloacae* C) *E. coli* D) *Klebsiella pneumoniae* E) *Pseudomonas aeruginosa* F-M) Gram-positive pathogens F) *Cutibacterium acnes* (Disease) G) *Cutibacterium acnes* (Healthy) H) *Enterococcus faecalis* I) Group A *Streptococcus* J) Methicillin resistant *Staphylococcus aureus* K) Methicillin sensitive *Staphylococcus aureus* L) *Staphylococcus epidermidis* M) vancomycin resistant *Enterococcus faecalis.*

**Supplemental Figure 3: Iodine skin ointment is highly effective at killing Gram-negative and Gram-positive pathogens.** A-M) Percent CFU reduction after 15, 30, or 60 seconds contact time with iodine skin ointment. Iodine skin ointment was serially diluted two fold in a 96 well plate and ~10^5^ CFUs of washed bacterial suspension were added. After 15, 30, or 60 seconds, 50 mg/mL sodium thiosulfate was added to neutralize iodine. Wells were plated in triplicate for enumeration. Percent CFU calculated relative to untreated conditions. All graphs representative of technical replicates from three independent experiments ± SEM. A-E) Gram-negative bacterial pathogens A) *Acinetobacter baumannii* B) *Enterobacter cloacae* C) *E. coli* D) *Klebsiella pneumoniae* E) *Pseudomonas aeruginosa* F-M) Gram-positive pathogens F) *Cutibacterium acnes* (Disease) G) *Cutibacterium acnes* (Healthy) H) *Enterococcus faecalis* I) Group A *Streptococcus* J) Methicillin resistant *Staphylococcus aureus* K) Methicillin sensitive *Staphylococcus aureus* L) *Staphylococcus epidermidis* M) vancomycin resistant *Enterococcus faecalis.*

**Supplemental Figure 4: Iodine skin ointment is highly effective at killing fungal pathogens.** A-M) Percent CFU reduction after 15, 30, or 60 seconds contact time with iodine soak, nasal antiseptic, and skin ointments. Iodine products were serially diluted two fold in a 96 well plate and ~10^5^ CFUs of washed fungal suspension were added. After 15, 30, or 60 seconds, 50 mg/mL sodium thiosulfate was added to neutralize iodine. Wells were plated in triplicate for enumeration. Percent CFU calculated relative to untreated conditions. All graphs representative of technical replicates from three independent experiments ± SEM. A-D) Iodine soak E-H) Nasal antiseptic I-L) Skin ointment A, E, I) *Aspergillus brasiliensis* B, F, J) *Aspergillus fumigatus* C, G, K) *Candida albicans* D, H, K) *Candidozyma auris*

|  | **Soak** | **Nasal Antiseptic** | **Skin Ointment** |
| --- | --- | --- | --- |
| ***Acinetobacter baumannii*** | 42.1875 | 70.3125 | 4.21875 |
| ***Enterococcus cloacae*** | 42.1875 | 70.3125 | 4.21875 |
| ***Escherichia coli*** | 42.1875 | 70.3125 | 4.21875 |
| ***Klebsiella pneumoniae*** | 42.1875 | 70.3125 | 4.21875 |
| ***Pseudomonas aeruginosa*** | 42.1875 | 70.3125 | 4.21875 |
| ***Cutibacterium acnes* (Disease)** | 42.1875 | 70.3125 | 4.21875 |
| ***Cutibacterium acnes* (Healthy)** | 42.1875 | 70.3125 | 4.21875 |
| ***Enterobacter faecalis*** | 84.375 | 70.3125 | 8.4375 |
| **Group A *Streptococcus*** | 84.375 | 70.3125 | 8.4375 |
| **MRSA** | 42.1875 | 70.3125 | 4.21875 |
| **MSSA** | 42.1875 | 70.3125 | 4.21875 |
| ***Streptococcus epidermidis*** | 42.1875 | 70.3125 | 4.21875 |
| **VRE** | 42.1875 | 70.3125 | 8.4375 |
| ***Candidozyma auris*** | 42.1875 | 70.3125 | 4.21875 |
| ***Candida albicans*** | 42.1875 | 70.3125 | 8.4375 |
| ***Aspergillus fumigatus*** | 42.1875 | 140.625 | 8.4375 |
| ***Aspergillus brasiliensis*** | 168.75 | 140.625 | 16.875 |

**Supplemental Table 1: Minimum concentration required for >99.9% killing.** Summary of minimum bactericidal or fungicidal (MBC, MFC) concentration in parts per million (PPM) required to kill >99.9% of starting inoculum for all strains tested.
